# Lineage B Genotype III of Dengue Virus Serotype 3 (DENV-3III_B) Is Responsible for Dengue Outbreak in Dire Dawa City, Ethiopia, 2023

**DOI:** 10.3390/v17030346

**Published:** 2025-02-28

**Authors:** Abebe Aseffa Negeri, Dawit Hailu Alemayehu, Saro Abdella Abrahim, Tsigereda Kifle Wolde, Gutema Bulti Tura, Alemnesh Hailemariam Bedasso, Danile Tsega Geretsion, Ebise Abose Djirata, Eyilachew Zenebe Awule, Diana Rojas-Gallardo, Asefa Konde Korkiso, Kalkidan Melaku, Raffael Joseph, Abaysew Ayele, Mesfin Mengesha Tsegaye, Anne Piantadosi, Getachew Tollera, Alemseged Abdissa, Mesay Hailu Dangiso, Adane Mihret, Andargachew Mulu, Tesfaye Gelanew

**Affiliations:** 1 Ethiopian Public Health Institute, Addis Ababa P.O. Box 1242, Ethiopia; 2 Armauer Hansen Research Institute, Addis Ababa P.O. Box 1005, Ethiopia; 3 Dire Dawa City Administration Health Bureau, Dire Dawa P.O. Box 1377, Ethiopia; 4 Population Biology, Ecology and Evolution Graduate Program, Emory University, Atlanta, GA 30322, USA; 5 Department of Pathology and Laboratory Medicine, Emory University School of Medicine, Atlanta, GA 30322, USA

**Keywords:** DENV, DF, serotype, genotype, CprM, DENV-3/GIII, Dire Dawa City, Ethiopia

## Abstract

The eastern parts of Ethiopia, including Dire Dawa City, have experienced annual dengue fever (DF) outbreaks since 2013, leading to significant healthcare and economic impacts. However, comprehensive evidence on the specific dengue virus (DENV) serotypes and genotypes involved remains limited. During the 2023 DF outbreak, the National Arbovirus Laboratory received seventy serum samples from suspected DF patients. Positive samples underwent sequencing of the CprM region of the DENV genome, and the obtained sequences were analyzed phylogenetically. Among the patients, 32 (45.7%) displayed early warning signs of severe dengue, and 13 were hospitalized, most showing symptoms indicative of severe dengue. Out of 67 adequate samples, 44 (65.6%) tested positive for DENV RNA by RT-PCR, and 17 successfully underwent CprM sequencing. All sequenced samples were identified as DENV-3, genotype III, major lineage B (DENV-3III_B), with two distinct minor lineages (DENV-3III_B.2 and DENV-3III_B.3). Phylogenetic analysis showed that these lineages were closely related to sequences from the Afar region, suggesting interconnected outbreaks with multiple co-circulating lineages. This study identifies DENV-3III_B as the cause of the 2023 DF outbreak in Dire Dawa City and highlights the need for enhanced viral genomic surveillance in Africa.

## 1. Introduction

Dengue fever (DF), caused by four genetically distinct serotypes of dengue virus (DENV-1 to DENV-4), has become a significant global health challenge. Over the past two decades, the incidence of dengue has increased tenfold, rising from 500,000 reported cases worldwide in 2000 to a staggering 5.2 million cases in 2019. In 2019, dengue outbreaks spread across 129 countries, marking an unprecedented peak. Although there was a slight decline in cases between 2020 and 2022 due to the COVID-19 pandemic and reduced reporting rates, 2023 witnessed a resurgence in dengue cases globally [1,2,3].

In areas where DF cases have been identified as endemic and/or epidemic, especially in tropical and subtropical countries, nonspecific febrile illnesses are common. Febrile illnesses can be caused by a variety of infectious agents such as malaria parasites, alphaviruses, and flaviviruses, complicating surveillance and response programs for outbreaks and endemic diseases [4]. In addition, the Aedes mosquito species, particularly *Ae. aegypti* and *Ae. albopictus* that transmit DENV, is also responsible for the transmission of other arboviruses (e.g., Zika virus, Yellow Fever virus, and Chikungunya virus), and is common in tropical and subtropical countries [5].

**Figure 1:**
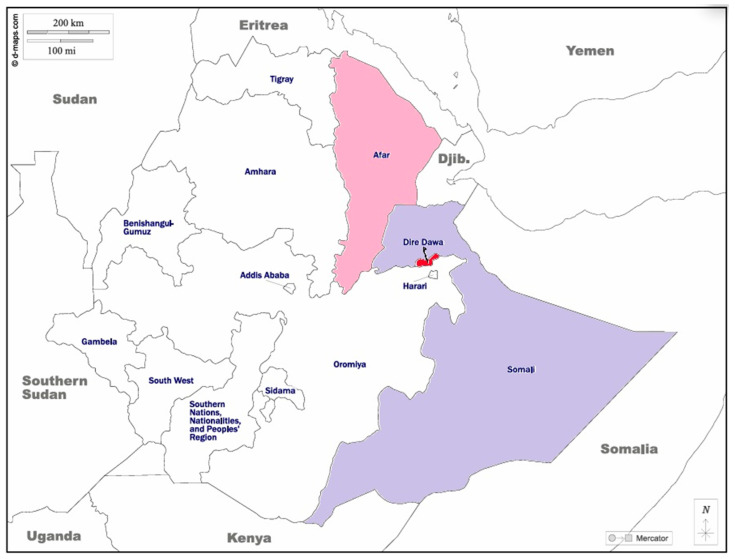
Map showing the Dire Dawa City Administration where the Dengue outbreak occurred, and the study of DENV strains originated. The shaded areas indicate the epicenters of dengue outbreaks in Ethiopia since 2013: Dire Dawa (red), Afar (red-violet), and Somalia (light blue).

In Ethiopia, the first laboratory-confirmed outbreak of DF occurred in Deri Dawa City in 2013. Since then, Ethiopia has experienced nearly annual outbreaks of DF in multiple regions of the country (Figure 1). In the Afar region, DF outbreaks occurred in 2019 in the Gewane District [6]. In the Ethiopian Somalia region, the first outbreak was reported in Godey town between 2014 and 2015 followed by outbreaks that occurred in Kabridahar in 2017 [7] and Warder Woreda in 2021 [8]. These recurrent outbreaks have impacted an already fragmented health system [9] and the economy of these parts of the country.

In April 2023, an outbreak of DF was initially reported in the Afar region, the northeastern part of Ethiopia. The first affected districts were Logia and Mille. Since April 4, 2023, however, it has spread to all seven districts and towns in the Afar region (Figure 1). As of 26 June 2023, a total of 6133 suspected and confirmed cases were reported, resulting in nine associated deaths (with a case/fatality ratio of 0.5%) [10]. It was documented that 8 of the 10 epidemic samples examined by reverse transcriptase polymerase chain reaction (RT-PCR) triplex [for Dengue, Zika, and Chikungunya] belonged to serotype DENV-3 [10].

As a suspected expansion of the outbreak in the Afar region, a DF outbreak was recorded in Dire Dawa City in December 2023 (Figure 1). However, there are no data regarding the DENV serotypes and genotypes associated with the outbreak. Whether the outbreak was caused by DENV-3 as in the Afar region remains unknown. In the present study, we present data on the serotypes, genotypes, and lineages of the DENV strains responsible for the outbreak in Dire Dawa City during 2023 and discuss the epidemiological and clinical consequences.

## 2. Materials and Methods

### 2.1. Ethical Considerations

As our study aimed to better understand DENV strains linked to DF outbreaks, we obtained an ethical approval waiver (protocol number: PO-036-24) from the All-African Leprosy Rehabilitation and Training Center/Armauer Hansen Research (ALERT/AHRI) Ethics Committee. Additionally, we obtained permission from the Dire Dawa City Health Bureau and health facilities’ authorities to investigate these outbreak samples.

### 2.2. Study Settings and Patient Data

**Figure 2:**
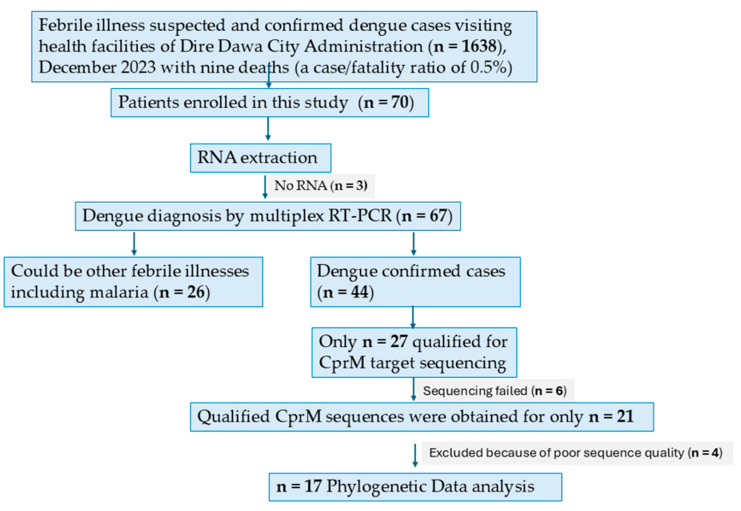
Flow chart showing enrollment of suspected dengue and/or other febrile illnesses, including malaria patients, and molecular detection and characterization of strains isolated during the 2023 dengue outbreak in Dire Dawa, Ethiopia.

On 14 December 2023, the Ethiopia Public Health Institute (EPH) received 70 serum samples from an outbreak of DF in Dire Dawa City Administration (Figure 1 and Table 1). These samples were collected from DF-suspected cases and shipped to the National Arbovirus Laboratory of EPHI for laboratory confirmation of the causative agent as part of an outbreak response investigation (Figure 2). The reported clinical signs and symptoms for each suspected case were extracted from medical charts available at the health facilities they were admitted to, using a case-based surveillance request form. Mild symptoms include sudden onset of high fever, vomiting, nausea, rash, arthralgia (joint pain), headache, chills, myalgia (muscle pain), sore throat, dizziness, runny nose, pruritus (itchiness), adenosis, diarrhea, and shivering. Warning signs indicative of severe dengue include belly pain or tenderness, persistent vomiting (at least 3 times/24 h), bleeding from the nose or gum, being restless or irritable, feeling weak, conjunctiva congestion, and convulsions (Table 2). The categories of mild symptoms and warning signs indicative of severe dengue are based on the WHO severe dengue classification [11].

### 2.3. Blood Sample Collection and Processing

Each of the 70 dengue-suspected patients submitted blood samples for laboratory diagnosis of the causative agent for febrile illness. The blood samples were allowed to clot, and the sera were separated. The sera were then shipped in a triple package cold chain to the National Arbovirus Laboratory of the EPHI. At the laboratory, the sera were kept frozen at −20 °C until they were used for DENV viremia detection using reverse transcriptase (RT)- PCR (RT-PCR).

### 2.4. Viral RNA Extraction and PCR Detection of DENV

Total RNA was isolated from each serum sample using the QIAmp viral RNA kit (QIAGEN, Hilden, Germany). The RNA was eluted in 50 μL of AVE buffer, which was provided with the kit. DENV viremia detection was conducted using RealStar^®^ Dengue RT-PCR kit (Altona Diagnostics, Hamburg-Altona, Germany). Briefly, 20 µL of the master mix (containing primers and probes) was mixed with 10 µL of the RNA sample or control. The mixture was centrifuged for 30 s at approximately 1000× *g* (~3000 rpm). The reactions were incubated for 20 min at 55 °C, followed by 40 cycles of 95 °C for 1 min, 55 °C for 45 s, 72 °C for 15 s, and final incubation at 68 °C for 10 min. The PCR results were interpreted based on the manufacturer’s protocol. A threshold cycle (Ct) value of ≤36 for DENV-specific RNA and a Ct value of ≤30 for the Internal Control were considered positive samples for DENV.

### 2.5. Semi-Nested RT-PCR Amplification (Amplicon Size 511 bp)

RNA samples with a Ct value of 31 or lower and sufficient volume were selected for CprM RT-PCR amplification following the procedure described elsewhere [12]. Briefly, the DENV CprM region (652) bp was first amplified using forward primer (FP): 5′-TCAATATGCTGAAACGCGCGAGAAACCG-3′ (132–153), the reverse primer (RP): 5′-GCGCCTTCNGNNGACATCCA-3′ (764–783), and the high-capacity cDNA reverse transcription kit (Invitrogen, Waltham, MA, USA). The reactions were incubated first at 98 °C for 10 s followed by 35 cycles of 98 °C for 10 s, 60 °C for 20 s, 72 °C for 30 s, and final incubation at 72 °C for 5 min. Semi-nested PCR was also used to amplify 511 bp of the CprM region of the viral genome using the amplified product as a template, and the same FP: 5′-TCAATATGCTGAAACGCGCGAGAAACCG-3′ (132–153) but a different RP-: 5′-TTGCACCAACAGTCAATGTCTTCAGGTTC-3′ (614–642) [12]. The semi-nested amplification was performed in an Applied Biosystems Veriti 96-Well Thermal Cycler (ThermoFisher Scientific, Waltham, MA, USA) using the following cycling parameters: 94 °C for 2 min; 40 cycles of 94 °C for 30 s, 70 °C for 1 s (ramp rate 20%), 55 °C for 45 s (ramp rate 20%), and 65 °C for 3 min, 20 s; and final extension for 10 min at 65 °C [13]. Following the amplification, 5 μL of the first amplicon and semi-nested amplicon size of 652 bp and 511 bp, respectively, was visualized on ethidium bromide-stained agarose gel under UV light. Amplified PCR products (511 bp in size) were then purified using a QIAquick PCR Purification Kit (QIAgen, Germany) and sequenced in both strands using a Big Dye Terminator Cycle Sequencing kit (Applied Biosystems, Waltham, MA, USA) with the same primers that were used for nested PCR. The CprM sequences were confirmed by BLAST. The forward and reverse sequences were aligned and manually edited using Bio Edit software (version 7.2) to obtain the consensus sequence for each sample, resulting in a 405 bp sequence spanning nucleotides 245–649 compared to reference sequence NC_001475. The obtained sequences were analyzed using Dengue Virus Typing Tool at Genome Detective server [13] to identify the serotypes, genotypes, and lineages of DENV associated with the outbreak.

### 2.6. Phylogenetic Analysis

Although CprM sequences were obtained for 21 DENV strains from Dire Dawa City and were of sufficient quality to determine serotype and genotype, only 16 consensus sequences were used for lineage and sublineage analysis with the specified tool. This was because 5 strains were excluded due to incomplete forward or reverse sequences and poor-quality chromatograms, which made relying on single-strand sequences impractical. The CprM consensus sequences for these 16 DENV strains from Dire Dawa City have been deposited in GenBank under accession numbers PP751832, PP751836-PP751845, and PP7547-PP751852. All DENV-3 CprM sequences available in the NCBI Virus database (https://www.ncbi.nlm.nih.gov/labs/virus/vssi/#/; accessed on 31 January 2025) were downloaded and aligned. Duplicates and identical sequences from the same country and collection date were removed. Representative sequences for DENV-3 genotypes I, II, and V were also included as references. The final data set includes a total of 942 DENV-3 CprM gene sequences collected between 2006 and 2023 across the globe.

For phylogenetic reconstruction, sequences were aligned using MAFFT (version 7.520) [14], and a maximum likelihood tree was constructed with the IQ-TREE software (version 2.2.2.7) [15] using the GTR+F+I+R3 substitution model (according to AICc) with a bootstrap value of 1000. Finally, the resulting tree was visualized and annotated using iTOL (Interactive Tree of Life) [16].

## 3. Results

### 3.1. Patient Characteristics

Among the 70 suspected dengue fever (DF) patients, 38 (54%) were female, as indicated in Table 1. The median age was 30 with an interquartile (IQ) range of 17 years. Most (81%) of the suspected cases visited health facilities as outpatients (Table 1), while the remaining 13 cases required inpatient hospitalization for a duration of 2 to 10 days.

The clinical signs/symptoms observed in these (n = 70) DF-suspected cases, whether admitted as outpatients or inpatients, covered a wide range of symptoms consistent with febrile illness. Nearly all of them exhibited three or more clinical symptoms consistent with mild DF. However, regardless of hospitalization status, 32 (45.7%) of these patients displayed one or more early warning signs indicative of severe dengue [11]. Among the 13 patients who were hospitalized for 2 to 10 days, all except two had at least one symptom indicative of severe dengue. The most commonly observed clinical presentations were acute fever (n = 66; 98.15%), headache (n = 65; 97.01%) and chills (n = 47; 70.17%) followed by aches and pains (n = 31; 46.27%) and vomiting/nausea (n = 31; 46.27%) (Table 2). In addition, all of the cases with early warning signs of severe dengue were later confirmed to harbor DENV viremia using RT-PCR.

### 3.2. DENV Viremia Detection

Among 70 specimens, 3 samples failed to pass the DENV nucleic acid amplification quality control. Among the 67 samples that passed quality control, 65.67% (44/67) had DENV RNA detected (Figure 2).

### 3.3. DENV Sequence Classifications

Based on rRT-PCR CT < 31, 27 samples out of 44 DENV viremia-positive samples were selected for CprM sequencing, and 21 were successful (Figure 2) allowing classification of their serotypes, genotypes, and lineages using a newly developed lineage system tool (https://dengue-lineages.org) [13].

**Figure 3:**
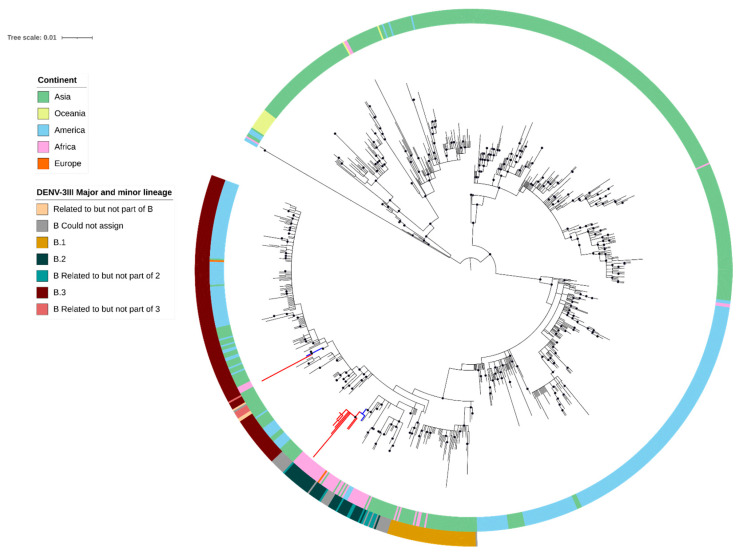
Maximum likelihood phylogenetic tree of DENV-3 genotype III (DENV-3III) strains isolated during the 2023 outbreak in Dire Dawa, Ethiopia. The tree includes CprM sequences newly generated in this study (red branches, N = 17) and representative reference sequences from DENV-3III (N = 942, sequences from Ethiopia in blue and other branches in black). The outer circles indicate continent of origin and the DENV-3III_B major and minor lineages. Nodes with ultrafast bootstrap values of 95 and above are indicated with black circles. The internal tree scale represents the number of nucleotide substitutions per site. A more detailed tree with branch labels, including accession number, country of origin, and year of isolation, as well as the percentages of support for the strains’ genotype, major lineage, and minor lineage assignments using recently developed DENV lineage assignment tools, can be found in Supplementary Figure S1 and Supplementary Table S1, respectively.

The sequence quality for five samples was insufficient, and these sequences were excluded from the DENV minor lineage analysis using the new dengue virus typing tool. According to this newly proposed DENV nomenclature [13], the dengue virus typing tool classified all 942 DENV-3 CprM gene sequences collected between 2006 and 2023, including those from our study, as DENV-3II. These sequences were further divided into minor lineages: DENV-3III_B.1, DENV-3III_B.2, and DENV-3III_B.3. All these minor lineages of DENV-3III_B were also found among sequences from the African continent, although some sequences were not assigned to specific minor lineages by the tool. Notably, the new tool assigned all 17 of our sequences to DENV-3, genotype III, major lineage B. Specifically, the minor lineages were assigned as follows: 1 sequence (DENV-3-1357) belonged to minor lineage 3 (DENV-3III_B.3), and 15 sequences (DENV-3-1355, -1363, -1395, -1399, -1400, -1401, -1403, -1404, -1405, -1410, -1411, -1412, -1414, -1415, and -1416) belonged to minor lineage 2 (DENV-3III_B.2). Nonetheless, the new lineage assignment tools did not assign one sequence (DENV-3-1402) to a minor lineage. This sequence was found to be related to minor lineage B2, but not part of it. Although minor lineages 1 (B.1), 2 (B.2), and 3 (B.3) have been documented in DENV 3 lineage III [13], the DENV strains investigated in our study were not assigned to minor lineage 1 (B.1) based on the classification determined by the recently developed dengue virus tool (Figure 3 and Supplementary Figure S1). Supplementary Table S1 presents the 17 DENV strains for which high-quality CprM sequences were obtained and deposited into the GenBank database repository, along with their corresponding accession numbers, serotypes, genotypes, linages, and minor lineages—assigned to them by the newly developed lineage assignment tools [13].

### 3.4. Phylogenetic Tree

We investigated the phylogenetic relationships between the 17 new DENV-3III_B CprM sequences obtained in this study and other DENV-3III_B CprM sequences from various regions, including Africa (Kenya, Gabon, Burkina Faso, Mozambique, Tanzania, and Ethiopia), Asia (the Middle East, India, Sri Lanka, Thailand, and China), the Americas (Jamaica, Martinique, Dominican Republic, and the USA), and Europe (Italy), deposited in a public sequence repository (DDBJ/ENA/GenBank-INSDC). As expected, the 15 DENV-3III_B minor lineage 2 (DENV-3III_B.2) sequences, including the one related to B.2, clustered separately from the single DENV-3III_B minor lineage 3 (DENV-3III_B.3) sequence (Figure 3). Both the DENV-3III_B.2 and DENV-3III_B.3 sequences from this study clustered closely with reference sequences isolated from the 2023 outbreak in the Afar region of Ethiopia [17], supporting the hypothesis that the outbreak in Dire Dawa was related to the outbreak in the Afar region, and indicating that multiple lineages co-circulated in both outbreaks.

## 4. Discussion

Knowledge of the genetic diversity of DENV strains responsible for outbreaks is essential for efficient management, vaccine development, and outbreak prevention. Though Ethiopia’s eastern region, including Dire Dawa City, has seen many DF outbreaks since 2013, there is scant information regarding the genetic diversity of circulating DENV strains. In this study, we used genetic analysis of the DENV CprM region to demonstrate that the 2023 outbreak in Dire Dawa City was due to two co-circulating lineages of DENV-3III_B, each of which was closely related to sequences from a related outbreak in the Afar region of Ethiopia in 2023. Our results are consistent with a recent study that analyzed 7 full DENV-3III genome sequences from the Afar outbreak [17], supporting the hypothesis that the outbreak in Dire Dawa was related to the outbreak in the Afar region, and indicating that multiple lineages co-circulated in both the Afar and Dire Dawa outbreaks. However, the paucity of regional DENV sequence data makes it difficult to determine the timing and origin of these introductions. Recently, other DENV-3III outbreaks have been reported in East Africa, notably in South Sudan in 2022 [18] and in Kenya in 2019 [19]. We were not able to assess the genetic relatedness between the current strains and outbreak strains from these neighboring countries due to the fact that they employed other targets for sequencing, such as the E gene. These results underscore the need for further genomic surveillance of DENV in East Africa, ideally using full-length genome sequences.

In contrast to the prior dengue outbreaks in Ethiopia which were mild [7,20,21,22], in this study, 46% (n = 32) of the 70 DF-suspected cases displayed early warning signs indicative of severe dengue, while the remaining cases showed mild symptoms. All of these cases with early warning signs of severe dengue were confirmed to harbor DENV viremia using RT-PCR.

We propose three scenarios that might have led to the occurrence of severe cases of DF during the 2023 outbreak in Dire Dawa City. One possibility is that the observed severe dengue cases could be secondary DENV-3III infections that happened after primary infection with a different serotype. In support of this, prior DF outbreaks reported from Dire Dawa City were associated with DENV-1 and DEV-2 strains [7,20,21,22,23], although previous attempts to determine serotypes were restricted to a small number of DENV strains. Thus, the recurrent occurrence of dengue outbreaks associated with different DENV serotypes highlights the need for close monitoring and management of future DF outbreaks to effectively control and manage secondary infections.

The second possibility is that DENV-3III is more pathogenic, as suggested by prior research that connected outbreaks of DENV-3III to Dengue Hemorrhagic Fever (DHF) and Dengue Shock Syndrome (DSS) [24].

The third possibility is that the severe cases may be the result of co-infection with malaria parasites, as both infections are co-endemic in Dire Dawa City, and there is a chance that co-infection can worsen disease severity and outcomes. This is supported by the recent surge in malaria [25,26] and dengue cases [27,28] in Dire Dawa City, which posed significant challenges for healthcare providers. Given the co-endemicity and overlapping of symptoms (e.g., fever), there is a risk of co-infection with both dengue and malaria and misdiagnosing dengue as malaria (and vice versa). Thus, an integrated approach involving surveillance, education, and differential diagnosis is crucial for better patient care in Dire Dawa City Administration. Healthcare professionals need to be aware of the clinical features, diagnostic tests, and management protocols for malaria and dengue. In addition, the Dire Dawa City Administration should ensure that appropriate laboratory tests (such as rapid diagnostic tests and PCR) are available in all health facilities to enable accurate diagnosis of dengue and malaria and prompt treatment and management of severe cases.

Among the five distinct genotypes (I–V) of DENV-3, all 21 sequenced DENV strains from the present study belonged to DENV-3III, which is the most widespread and was associated with large outbreaks in Asia, Africa, and the Americas [29,30,31]. The first autochthonous case of DENV-3III in Africa was reported in Mozambique in 1985 [32]. Several man-made and natural factors, including (i) migration and conflict-related population displacement, (ii) frequent migration via trade routes that can spread the virus to new areas, and (iii) recurrent flooding that is made worse by climate change that provides ideal breeding grounds for Aedes mosquitoes, may have contributed to the rapid geographic expansion of DENV-3III in Africa, resulting in outbreaks in areas like Dire Dawa City [3,24,29,33,34].

Even though this work offers important insights into DENV strains responsible for the 2023 DF outbreak in Dire Dawa City where severe cases and deaths were recorded, it suffers limitations. First, the study’s capacity to detect genetic variation was limited due to the use of a short genomic target (CprM, 511 base pairs) for the characterization of the outbreak strains. Expanding the genomic coverage could have enhanced our understanding of DENV diversity and relationships to other DENV-3III strains across the globe. Second, DENV CprM sequencing was performed on a small proportion of DF-suspected cases reported during the outbreak, and a larger sample size may have identified greater genetic diversity. Third, the study lacks information on other febrile illness-causing pathogens like malaria parasite examination with microscopy or rapid diagnostic test results for the studied DF-suspected cases, so cases of co-infection may have been missed. Future research addressing these limitations will therefore improve our comprehension of disease dynamics, provide a thorough understanding of DENV strains and their evolutionary history, expand our understanding of DENV outbreaks, and provide guidance for more effective preventative measures.

Historically, DENV infection and its severe manifestations have been underreported on the African continent [35]. However, this has changed recently. For instance, a study in Burkina Faso found that 33.5% of dengue cases were severe, with renal failure and severe bleeding being common complications [36]. Similarly, the present study reported a comparable proportion of dengue cases with warning signs that were predictors of severe dengue [37]. The severity and mortality of dengue infection in Africa may be associated with bacterial or other febrile coinfections (including malaria), comorbidities, or DENV serotypes [36,38]. However, the current study was hindered by the lack of crucial patient information, including immunosuppression, coinfections, and comorbidities, due to the unavailability of such data during the review of patient medical charts. Future studies should include comprehensive clinical and laboratory assessments of dengue patients to determine dengue fever severity.

## 5. Conclusions

In summary, our results demonstrate that the 2023 DF outbreak in Dire Dawa City, Ethiopia, was due to multiple co-circulating lineages of DENV-3III. The severe cases recorded during this outbreak may be due to either the genetic makeup of DENV-3III, secondary DENV infection, or co-infection of DENV and malaria. Additionally, the intercontinental transmission of DENV-3III underscores the importance of vigilant surveillance and preparedness to mitigate the impact of such outbreaks. Thus, collaborative efforts across borders are crucial for effective prevention and control strategies.

## Figures and Tables

**Table 1:** Status of admission and demographic characteristics of dengue fever suspected cases (n = 70) reported at health facilities in the Dire Dawa City Administration, 2023.

Variable	Specific Variable	N (%)
Age	Median age 30; IQR ^1^ 17	
Sex	Male	32 (46) ^2^
	Female	38 (54)
Patient admission status	Outpatient	57 (80)
	Inpatient	13 (20)

^1^ IQR = Interquartile range. ^2^ Values in parentheses indicate percentiles (%), while values before parentheses indicate the number (N) of participants.

**Table 2:** Clinical characteristics of dengue fever suspected cases (n = 70) reported at health facilities in the Dire Dawa City Administration, 2023.

Clinical Symptoms ^1^		Yes ^2^	No
**A.** **Mild symptoms** *	Fever: sudden onset of high fever	66 (98.15%)	1 (1.49%)
	Chills	47 (70.17%)	20 (29.85%)
	Vomiting or nausea	31 (46.27%)	36 (53.73%)
	Aches and pains: Typically, behind the eyes, muscle, joint, or bone pain	31 (46.27%)	36 (53.73%)
	Myalgia	28 (45.9%)	33 (54.1%)
	Running nose	21 (31.34%)	46 (68.66%)
	Diarrhea	13 (19.4%)	54 (89.60%)
	Rash	8 (13.11%)	53 (86.89%)
	Sore throat	6 (8.89%)	61 (91.04%)
	Dizzy	5 (7.46%)	62 (92.54%)
	Shivering	4 (5.97%)	63 (94.03%)
	Pruritus	2 (2.99%)	65 (97.01%)
	Adenoids	1 (1.52%)	65 (98.48%)
**B.** **Warning signs indicative of severe dengue**	Weak (Feeling tired)	26 (38.81%)	41 (61.19%)
	Restless or irritable	15 (22.39%)	52 (77.61%)
	Arthralgia	9 (13.43%)	58 (86.57%)
	Persistent vomiting (at least 3 times/24 h)	9 (13.43%)	58 (86.57%)
	Bleeding from the nose or gums	6 (8.96%)	61 (91.04%)
	Belly pain or tenderness	5 (7.46%)	62 (92.54%)
	Conjunctival congestion	3 (4.48%)	64 (95.52%)

^1^ Categorization of mild symptoms and warning signs indicative of severe dengue are based on WHO classification. ^2^ Clinical data for 4 cases out of the 70 suspected dengue fever cases were missing or incomplete. * Mild symptoms indicate a lower level of severity that does not pose an immediate threat to health. Conversely, warning signs indicate a severe dengue that could necessitate emergency intervention.

## Data Availability

The datasets generated and/or analyzed during the current study are available in the manuscript and the Supplementary Materials.

## Supplementary Materials

The following supporting information can be downloaded at: https://www.mdpi.com/article/10.3390/v17030346/s1, Supplementary Figure S1. Maximum likelihood phylogenetic tree of DENV-3 genotype III (DENV-3III) strains isolated during the 2023 outbreak in Dire Dawa, Ethiopia with branch labels (the accession number, country of origin, and year of isolation). Supplementary Table S1: The 17 DENV strains for which high-quality CprM sequences were obtained and deposited into the GenBank database repository, along with their corresponding accession numbers, serotypes, genotypes, linages, and minor lineages, assigned to them by the newly developed lineage assignment tools.
