# Supplementary material for: Lineage B Genotype III of Dengue Virus Serotype 3 (DENV-3III_B) Is Responsible for Dengue Outbreak in Dire Dawa City, Ethiopia, 2023
Source: Viruses. 2025 Feb 28;17(3):346. doi: 10.3390/v17030346 (PMC11945396; doi:10.3390/v17030346)
Supplement: Supplementary file 1 [file viruses-17-00346-s001.zip › viruses-3396937-supplementary/Viruses-Supplematry/Supplementary_Figure_1.V2 (1).pdf]

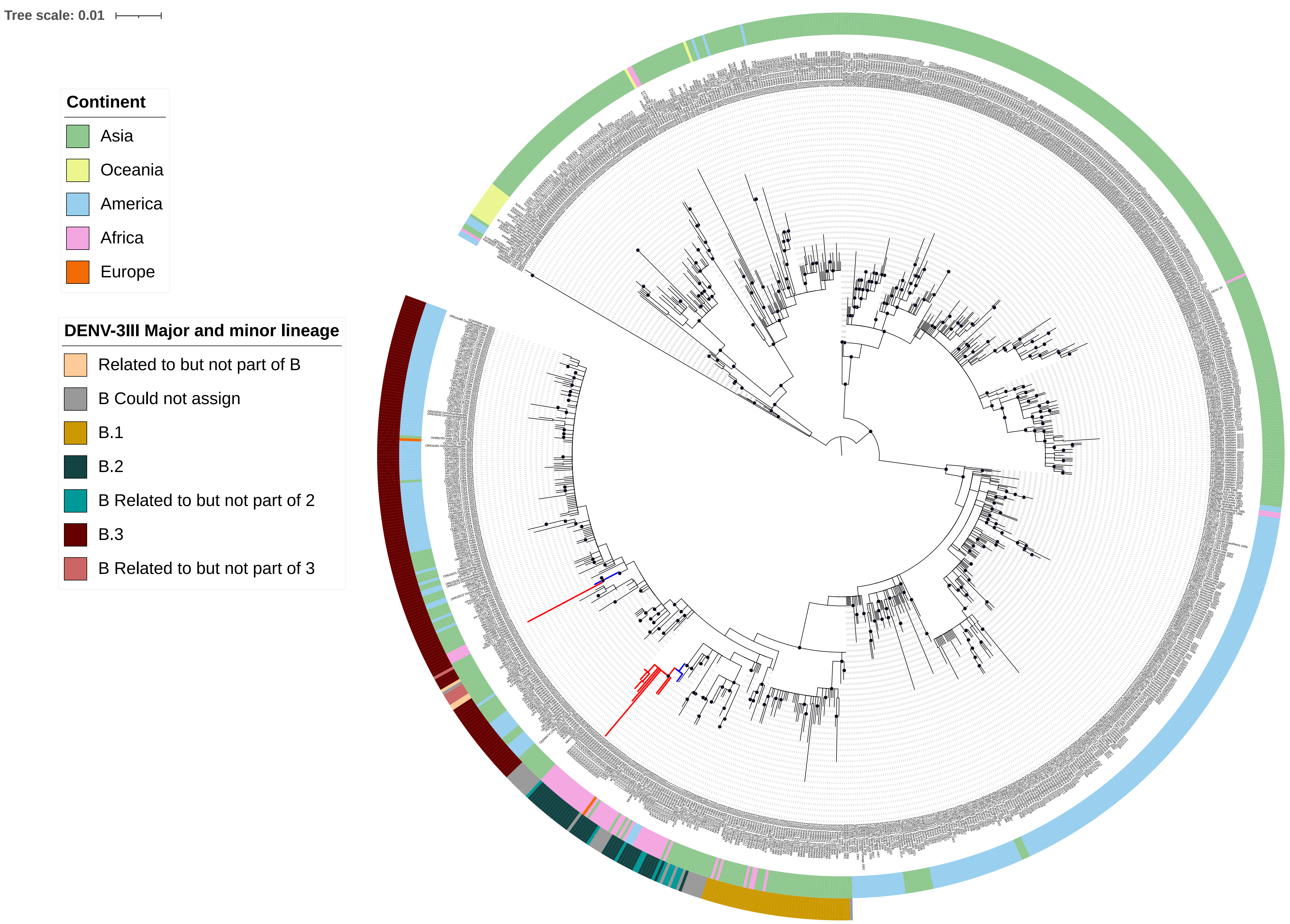

Supplementary Figure S1. Maximum likelihood phylogenetic tree of DENV-3 genotype III (DENV-3III) strains isolated during the 2023 outbreak in Dire Dawa, Ethiopia with branch labels. The tree includes CprM sequences newly generated in this study (red branches, N = 16) and representative reference sequences from DENV-3III (N = 942, sequences from Ethiopia in blue and other branches in black). Sequences are labeled with the accession number, country of origin, and year of isolation, when available. The outer circles indicate continent of origin and the DENV-3III_B major and minor lineage. Nodes with ultrafast bootstrap values of 95 and above are indicated with black circles. The internal tree scale represents the number of nucleotide substitutions per site.
